# Nonlinear-Based MEMS Sensors and Active Switches for Gas Detection

**DOI:** 10.3390/s16060758

**Published:** 2016-05-25

**Authors:** Adam Bouchaala, Nizar Jaber, Omar Yassine, Osama Shekhah, Valeriya Chernikova, Mohamed Eddaoudi, Mohammad I. Younis

**Affiliations:** Physical Sciences and Engineering Division, King Abdullah University of Science and Technology, Thuwal 23955-9600, Saudi Arabia; adam.bouchaala@kaust.edu.sa (A.B.); nizar.jaber@kaust.edu.sa (N.J.); omar.yassine@kaust.edu.sa (O.Y.); Osama.Shekhah@kaust.edu.sa (O.S.); valeriya.chernikova@kaust.edu.sa (V.C.); mohamed.eddaoudi@kaust.edu.sa (M.E.)

**Keywords:** microbeam, gas sensor, active switch, microcontroller, nonlinear dynamics, metal-organic framework

## Abstract

The objective of this paper is to demonstrate the integration of a MOF thin film on electrostatically actuated microstructures to realize a switch triggered by gas and a sensing algorithm based on amplitude tracking. The devices are based on the nonlinear response of micromachined clamped-clamped beams. The microbeams are coated with a metal-organic framework (MOF), namely HKUST-1, to achieve high sensitivity. The softening and hardening nonlinear behaviors of the microbeams are exploited to demonstrate the ideas. For gas sensing, an amplitude-based tracking algorithm is developed to quantify the captured quantity of gas. Then, a MEMS switch triggered by gas using the nonlinear response of the microbeam is demonstrated. Noise analysis is conducted, which shows that the switch has high stability against thermal noise. The proposed switch is promising for delivering binary sensing information, and also can be used directly to activate useful functionalities, such as alarming.

## 1. Introduction

Microelectromechanical systems (MEMS) are considered very attractive due to their outstanding capabilities and unprecedented performance for sensing applications. MEMS sensors have broad uses encompassing different domains, such as medicine, aerospace, automotive, public safety, and national security [1,2,3,4,5,6]. The importance of small mass detection and measurement has led to the development of sophisticated microstructure designs with very high sensitivity reaching femtogram resolution [7,8]. The need for accurate and highly sensitive microsensors has led to cutting-edge research in biology and chemistry to detect very small mass quantities [9,10].

MEMS sensors based on tracking the resonance frequency shift have been widely used in mass and gas sensing due to their higher sensitivity [11,12,13,14,15,16,17,18,19]. Burg *et al.* [14] developed a new design for a mass sensor to measure the weight of single cells and single nanoparticles in fluid. They designed a suspended microchannel on a microcantilever beam. A frequency shift is measured when the added mass reaches the tip. The structure is electrostatically actuated and vibrates in the vacuum. Fadel *et al.* [18] studied analytically and experimentally the signal-to-noise ratio of a chemical microsensor as a function of its resonance frequency and its quality factor. They demonstrated that the selection of a highly sensitive chemical sensor should be based on maximizing the ratio between quality factor and the microstructure thickness. Yang *et al.* [19] demonstrated very high sensitivity in real time for zeptogram (10^–21^ g) mass detection using ultra-high frequency nanoelectromechanical systems.

Recently, new sensing techniques have been proposed to enhance the performance of mass sensors, such as using the nonlinear behavior of microstructures and exploiting the frequency shift upon analyte adsorption under parametric excitation [20,21]. Younis *et al.* [22] studied new concepts for mass detection using electrostatically actuated structures based on nonlinear dynamics. They investigated microstructures’ vibrations near the primary and secondary resonance and proposed the idea of a switch triggered by added mass based on the pull-in phenomenon. Zhang *et al.* [23] proposed and experimentally validated a new mass sensing concept based on parametric excitation. They detected the added mass by measuring the frequency shift at the edge of the first order parametric resonance.

Stability issues in the nonlinear regime have also been studied [24,25,26,27]. Kacem *et al.* [26] investigated theoretically and experimentally a dynamic stabilization technique for electrostatically actuated nanoresonators. The method is based on exciting the primary and superharmonic resonances simultaneously. Also, Antonio *et al.* [27] demonstrated a new way to stabilize mechanical oscillators by using internal resonance, which is induced between two bending modes.

Kumar and co-authors [28,29] proved experimentally a bifurcation based microsensor using a piezoelectric cantilever beam oscillating near its first natural frequency. Kumar *et al.* [28] proposed a biological and chemical sensor using a microcantilever beam coated with a poly-4-vinylpyridine polymer layer based on this phenomenon. The main idea is when operating near one of the saddle-node bifurcations; the mass adsorbed on the surface of the resonator will downshift the frequency response curve, which leads to a sudden jump in amplitude. A complete model and analysis study has been done in [29] to validate the experimental results of the bifurcation-based gas sensor. A new technique to use the nonlinear response for mass and gas sensing has been highlighted in [30,31]. Hiller *et al.* [30] developed and experimentally validated a new technique based on feedback control to track the amplitude and the induced frequency shift in real time upon gas exposure. The fabricated microbeam is excited parametrically in air, and the nonlinear response has been utilized for dimethyl methylphosphonate (DMMP) detection.

Electrostatically actuated microstructures have been used extensively as sensors and actuators due to their simplicity and compatibility with CMOS technology. Howe *et al.* [32] are considered among the first to use electrostatically actuated microstructures coated with polymer for gas detection. They used a microbridge made of polycrystalline silicon. Tang *et al.* [33] used an electrostatically actuated polysilicon comb drive in order to actuate it in air. They reported a quality factor ranging between 20 and 180 and a natural frequency ranging from 18 kHz to 80 kHz. Also, the CMOS technology has been investigated widely for mass and gas detection. Bedair *et al.* [34] fabricated an in-plane microstructure for gas detection coated with polystyrene, which represents the sensitive layer, using an inkjet printer. They demonstrated higher sensitivity for acetone owing to its potential to dissolve organic chemicals.

Metal-organic frameworks (MOFs) has been considered a very promising sensitive layer compared to other porous materials and polymers for gas applications [35,36]. Due to its tunable pore size and chemical functionality, high surface area, and selectivity to some chemicals, researchers have been attracted by MOFs and their capabilities for chemical detection [37,38,39]. Khoshaman *et al.* [38] used for the first time the HKUST-1 MOF to detect volatile organic compounds. Electrospraying systems have been utilized to coat quartz crystal microbalances (QCMs). Isopropyl alcohol, tetrahydrofuran, and acetone have been investigated with the coated QCM. Sapsanis *et al.* [39] investigated a capacitive sensor coated with MOF thin films for volatile organic compounds and humidity detection. The successful application of porous MOFs for sensing requires the incorporation of signal transduction. At the micro scale, MOFs sensors based on mechanical signal transduction have been investigated recently by integrating them on piezoresistive micro-cantilevers [40].

The objective of this paper is to demonstrate, for the first time, the integration of a MOF thin film on electrostatically actuated microstructures to realize a switch triggered by gas and a sensing algorithm based on amplitude tracking. Electrostatically actuated clamped-clamped microbeams are fabricated and coated with HKUST-1 MOF. The proposed electromechanical switch can perform two functionalities; from one hand it can work as a sensor and track the change in frequency before the sudden change in amplitude. On the other hand, it can work as an electrical switch (or as an actuator), which will be activated upon gas adsorption beyond a certain threshold.

## 2. Fabrication Process

**Figure 1:**
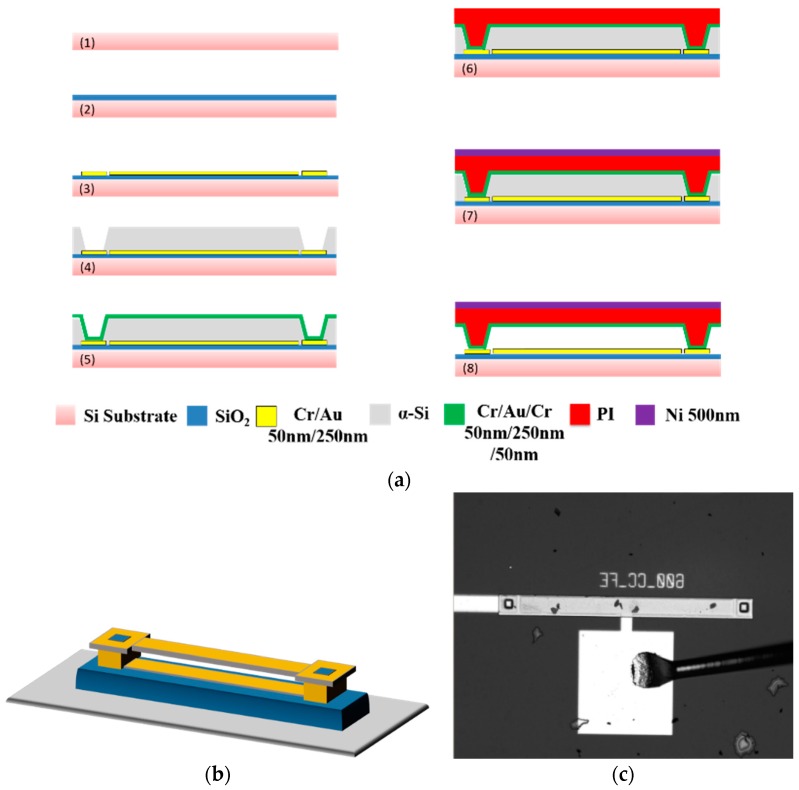
(**a**) The fabrication process flow of the clamped-clamped microbeam; (**b**) A schematic of an electrostatically actuated clamped-clamped microbeam; (**c**) An optical image of the fabricated microbeam.

Figure 1a–c represent the fabrication process flow, a schematic of the electrostatically actuated clamped-clamped microbeam and an optical image of the fabricated beam, respectively.

The clamped-clamped microbeam is fabricated using the fabrication process described in Figure 1a. The process starts with a 4-inch silicon wafer with a deposited silicon dioxide layer, which represents an insulation layer, Figure 1a (2). The first step is to sputter the lower electrode, which is composed of gold/chrome layer of thicknesses 250 nm/50 nm as shown in Figure 1a (3). Amorphous silicon is deposited on the top of the gold and chrome layers to form the sacrificial layer. Two anchors are etched in the amorphous silicon layer to connect the upper electrode with the lower connections as shown in Figure 1a (4). In Figure 1a (5), the upper electrode is fabricated with chrome/gold/chrome layers of thicknesses 50 nm/250 nm/50 nm. Then in Figure 1a (6), 6 μm of polyimide (PI) is spun and cured at gradually increasing temperature from 150 °C to 350 °C for 50 min and then held at 350 °C for 30 min to form the structural layer. Finally, in Figure 1a (7), a 500 nm nickel layer is sputtered on the top surface of the polyimide layer in order to protect the microbeam during the reactive ion etching, Figure 1a (8). The geometrical properties of the fabricated microbeam are shown in Table 1.

## 3. MOF and Inkjet Printing

**Figure 2:**
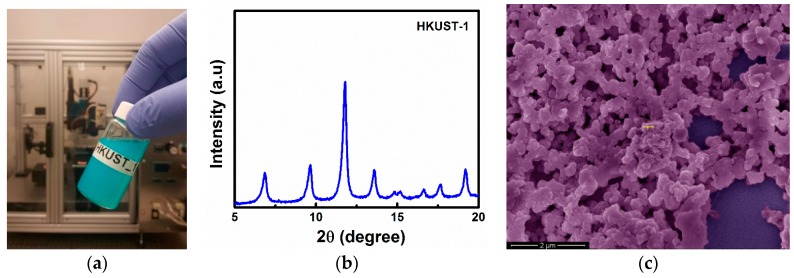
HKUST-1 metal-organic framework. (**a**) Photo of the MOF solution and the inkjet printer; (**b**) PXRD pattern and (**c**) Top-view SEM image of the HKUST-1 MOF thin film fabricated using the inkjet printing method.

MOFs are very promising porous materials for gas sensing applications [41,42,43,44], and therefore, the clamped-clamped microbeam was coated with a MOF thin film using an inkjet printer with a 20 μm diameter nozzle. In this work, we used Cu_3_(btc)_2_·xH_2_O MOF (where btc is 1,3,5-benzenetricarboxylate), also known as HKUST-1, which is prepared using the method described in [37]. The prepared HKUST-1 solution for inkjet printing has a light blue color, as shown in Figure 2a. The MOF solution is very stable and can be used even after several months [37]. The HKUST-1 MOF on the surface of the clamped-clamped microbeam was characterized using powder X-ray diffraction (PXRD) and scanning electron microscopy (SEM). Figure 2b,c confirm the proper formation of the targeted MOF thin film on the surface of the microbeam. A similar PXRD pattern has been demonstrated for HKUST-1 in the literature [44].

## 4. Gas Sensing Setup

**Figure 3:**
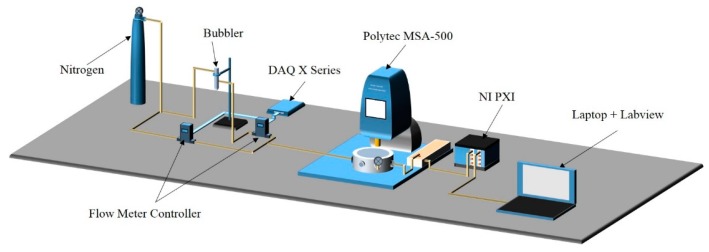
A schematic of the optical gas sensing setup.

In this section, we describe the gas sensing setup used for exposing the MOF coated microstructure to water vapor. Figure 3 shows a schematic of the setup with a high pressure nitrogen source connected from one side to the bubbler, which contains the desired gas (water vapor) in liquid phase, and connected from the other side to the flow meter controller to regulate the nitrogen flow when purging is needed to restore the frequency to its default value. The output of the bubbler is connected to a second flow meter to control the flow of the gas and the nitrogen mixture. A multifunction data acquisition card with the Labview program is utilized to control the flow rates. An environmental chamber is connected to the gas setup and is placed under the laser Doppler vibrometer from Polytec, Inc. (Mooresville, NC, USA) [45] in order to measure the microbeam deflection in real time.

The flow mass controller connected to the bubbler is set to allow 0.4 L/min and 0.1 L/min of flow for the jump-up and jump-down experiments, respectively. An inlet of the environmental chamber is connected to a pressure gauge to measure the pressure, which is equal to 3.3 Torr and 220 mTorr for the jump-up and jump-down experiments, respectively.

## 5. Sensor Characterization

Frequency response curves of the coated clamped-clamped microbeam are generated for various voltage loads. Here we recall that a clamped-clamped microbeam has two different sources of nonlinearities; the first one comes from mid-plane stretching due to the geometry of the structure and the fixed anchors. This nonlinearity is cubic and is dominant for low DC voltages, which leads to hardening behavior. The second source of nonlinearity is due to the electrostatic force, which is quadratic in nature, and leads to softening behavior [46]. Next, we show frequency response curves of the beam for two representative flow rates, and hence, damping conditions, at various voltage loads.

**Figure 4:**
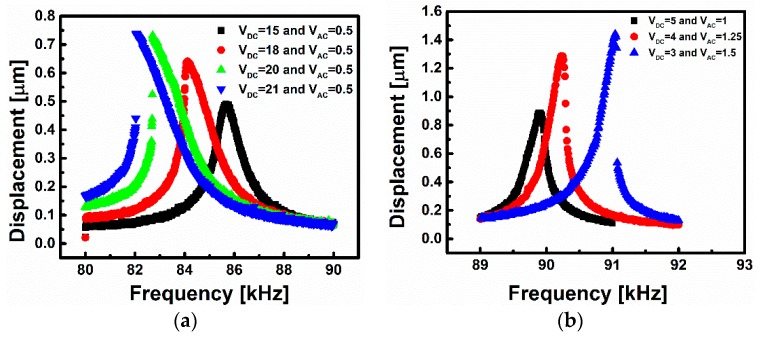
Frequency response curves, obtained through forward sweeps, of the clamped-clamped microbeam (**a**) for different DC voltages showing a transition from a linear to a softening behavior at 3.3 Torr; and (**b**) for different AC voltages showing the transition from linear to hardening behavior at 220 mTorr.

Figure 4a shows the frequency response curves of the microbeam for different DC values. The microbeam characterization has been performed at the same conditions of the gas sensing experiments, which is at a pressure of 3.3 Torr. One can note that the effect of pressure is crucial here since it can change the nonlinear behavior, which is needed for the proposed devices, into a linear behavior. In Figure 4a, the black-square curve represents the frequency response of the beam in the linear regime. Increasing the DC voltage, softening behavior starts to appear, and for some curves, this leads to hysteresis where two responses can exist at a single frequency. At *V_DC_* = 21 V and *V_AC_* = 0.5 V, a jump in the response of 0.3 μm occurs at the transition between the lower and upper dynamical states. This jump will be utilized for the switch triggered by gas. This type of switch will be called a jump-up switch. In Figure 4b, increasing the AC voltage with a lower range of DC voltage leads to the appearance of the hardening behavior (the electrostatic nonlinearity here is too weak). The advantage of a hardening frequency response curve is that for a certain voltage load an almost linear segment of the upper branch of the frequency-response curve appears before jumping to the second lower branch. This segment can be used and fitted to a linear equation to track the amount of mass attached on the surface of the microbeam. Almost 1 μm jump in amplitude has been shown in Figure 4b with *V_DC_* = 3 V and *V_AC_* = 1.5 V. This type of switch will be called a jump-down switch.

## 6. Results

In this section, the new switching technique based on the nonlinear response of the clamped-clamped microbeam is described. Mainly, we will discuss the two kinds of switches that have been introduced in the previous section. To determine the minimum detectable frequency, and hence, the minimum detectable mass of the proposed sensors and switches, we refer to the noise analysis presented in Appendix A.

### 6.1. Jump-Up Switch Triggered by Gas

Next, the jump-up switch mechanism is detailed based on humidity detection. Before starting the water vapor sensing measurement, we flush the microbeam with nitrogen for a long time to ascertain that the HKUST-1 MOF thin film is activated, and the solvent has been evaporated, in order to start from a stable frequency reference [47].

**Figure 5:**
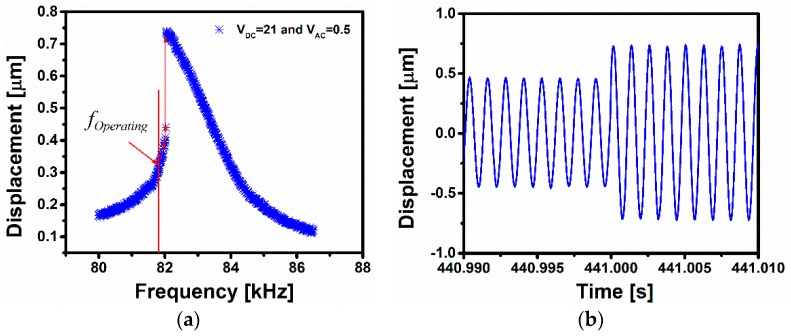
(**a**) Frequency response for the Jump-up switch indicating in the vertical red line the operating point of the device prior to mass detection. The red line highlights the jump in the response from the lower to the upper branches, which occurs upon mass detection when exceeding a certain threshold; (**b**) Time history of the beam displacement upon gas exposure showing the jump from the lower to the upper dynamical states, thereby triggering the switching event.

Figure 5a represents the frequency response for the jump-up switch for *V_DC_* = 21 V and *V_AC_* = 0.5 V. In Figure 5b, the time history of the mid-point of the microbeam close to the jumping regime has been performed upon vapor exposure at a pressure of 3.3 Torr. The frequency response curve is shifted to a lower range of frequency and then at a certain point the amplitude jumps-up suddenly. This abrupt change in amplitude is proposed to be used for triggering a switch. More details on the switch idea will be clarified while discussing the jump-down switch next.

### 6.2. Jump-Down Sensor and Switch

In addition to the jump-up switch based on the softening behavior for a high DC value, we demonstrate next the switching using the jump-down switch based on the hardening behavior of a clamped-clamped microbeam. The jump-down switch algorithm is the same used for the jump-up switch, which relies on the sudden change in amplitude. However, in this type of switch, one can quantify the amount of absorbed mass prior to the sudden jump or the switching event as will be detailed next.

In order to demonstrate the jump-down switch, a microcontroller from Arduino Inc. (Boston, MA, USA) [48] equipped with an ATMEGA328P microprocessor is connected to the gas sensing setup. The outcome from the DAQ is connected to the microprocessor. A Labview program with an Arduino library is developed in order to read the voltage coming from the laser Doppler controller at a fixed frequency. The algorithm is based on calculating the amplitude difference between two successive points during a frequency sweep. When the absolute value of the difference between the current and previous data point exceeds a defined constant, switching is triggered.

**Figure 6:**
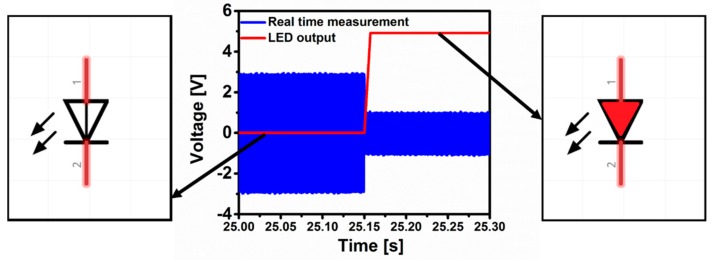
Time history of the sensor to trigger a LED upon water vapor exposure.

A LED is connected to the Arduino digital output in order to indicate the switching upon gas adsorption. As shown in Figure 6 the output voltage of the LED is tracked in red using a Labview program. When the jump occurs the voltage rises from 0 to 4.9 V, which represents the switching phenomenon.

**Figure 7:**
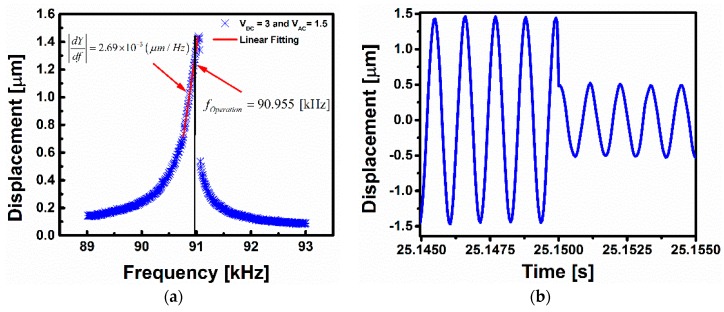
(**a**) Frequency response with the linear fitting of the upper branch at 220 mTorr. The linearly-fitted curve can be used to relate the amplitude change to the frequency shift, and hence, the amount of absorbed mass; (**b**) Time history of the beam displacement upon gas exposure indicating the sudden jump down, and hence, the switching event.

In Figure 7a, the nonlinear frequency response is shown for $V_{DC}=3\text{ V}$ and $V_{AC}=1.5\text{ V}$ under a pressure of 220 mTorr. Using this set of conditions, the cubic nonlinearity dominates and the hardening behavior appears in the frequency response. In the vapor detection results shown in Figure 7b, the same clamped-clamped microbeam used for the previous gas sensing with softening behavior has been utilized. However, the flow rate is set to be 0.1 L/min, which leads the pressure of the testing chamber to be equal to 220 mTorr.

**Figure 8:**
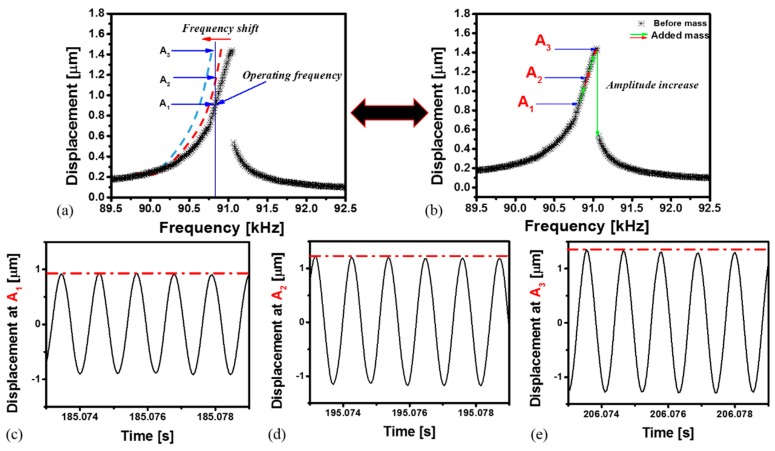
Mass quantification concept at different vapor exposure steps. (**a**) Frequency response curves before (experimental, black stars) and after vapor exposure (schematic, dashed). The vertical line indicates a fixed operating frequency of the resonator during vapor exposure. Clearly, the amplitude of the resonator increases with the absorption of vapor; (**b**) The same experiments in (**a**) can be viewed based on a single frequency-response curve before vapor exposure. Vapor absorption can be viewed as riding the upper branch of the frequency response curve; (**c**–**e**) Measured time history showing the displacement at (**c**) point A_1_; (**d**) point A_2_ and (**e**) point A_3_.

In Figure 8 we clarify an approximate technique to quantify the adsorbed mass from linearly fitting the upper branch of the frequency response curve in the hardening behavior. The concept relies on operating the resonator at a fixed frequency (*f_Operating_*) before the jump-down regime in the frequency response curve as shown in Figure 8a. Vapor exposure downshifts the frequency response curve. Hence the amplitude at the fixed operating frequency line in Figure 8a starts to increase, as illustrated through the different points of A_1_, A_2_ and A_3_. Figure 8a can be interpreted as an increase in amplitude along the linear branch of the frequency response, as shown in Figure 8b. Then, at the fixed frequency *f_Operating_*, the variation of the amplitude can be tracked as the microbeam is exposed to vapor as shown in Figure 8c–e. This change in amplitude can be quantified and related/calibrated to the amount of captured mass.

**Figure 9:**
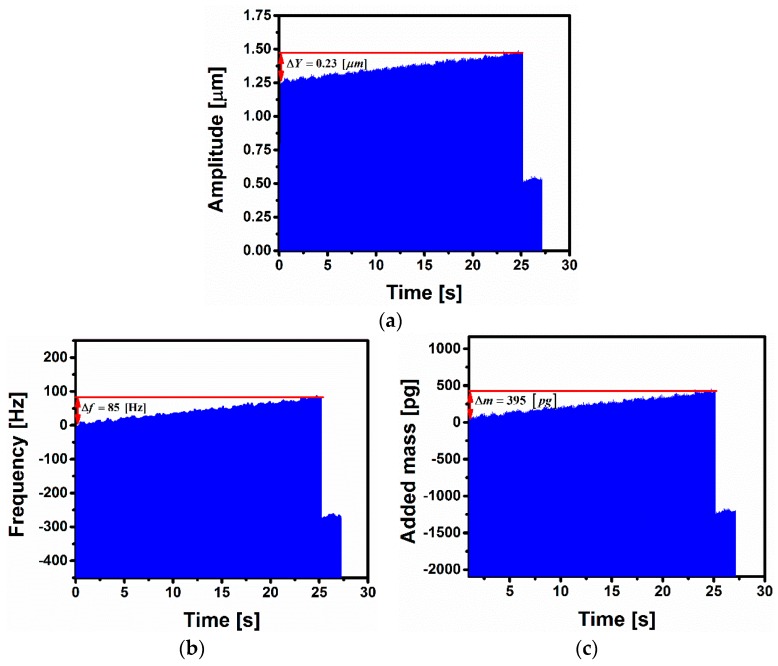
Time history of electrostatically actuated microbeam upon gas exposure (**a**) amplitude variation; (**b**) Frequency shift and (**c**) Added mass as a function of time.

Now referring back to Figure 7a, using a linear fitting, the slope of the linear branch is determined, which represents the variation of the amplitude with respect to the frequency $\left|dY/df\right|=2.69\times 10^{-3}\;\mu m/\text{Hz}$. Exposing the microbeam to vapor leads to an increase in amplitude. This is further clarified in Figure 9a, which shows a real time measurement of the microbeam mid-point deflection when exposed to water vapor. The amplitude of the fixed frequency *f_Operating_* = 90.955 kHz is equal to *Y_Operating_* = 1.25 μm before vapor exposure. After 25 s of vapor exposure, the amplitude of the point before jump (B-jump) reaches *Y_B-Jump_* = 1.48 μm, then it jumps-down, as shown in Figure 9a.

The frequency shift as a function of time is crucial information in the dynamic-based sensor to be determined. As the variation of amplitude has been done in the linearly fitted regime, the calculated slope is used to determine the frequency shift as a function of time shown in Figure 9b. We subtract the initial amplitude value from the amplitude variation and then we divide by the calculated slope. Measuring the frequency shift as a function of time from Figure 9b, we found Δ*f* = 85 Hz before reaching the jump zone. In order to check the accuracy of calculations, we compare the frequency shift coming from the real time measurement in Figure 9b with the frequency shift calculated from Figure 7a by subtracting the frequency of the point just before the jump *f_B-Jump_* = 91.04 kHz from the operating frequency *f_Operation_* = 90.955 kHz. The least calculated frequency is found to be equal to Δ*f* = 85.55 Hz, which is very close to the calculated frequency shift using linear fitting. Using Equation (2), the amount of the added mass Δ*m* can be tracked in real time from the induced frequency shift Δ*f* as shown in Figure 9c. The total mass attached on the sensor before the activation of the switch is $\Delta m={ℜ}^{-1}\Delta f=395\text{ pg}$.

## 7. Conclusions

In this paper, we demonstrate the advantage of using the nonlinear response of an electrostatically actuated resonator for gas sensing. A clamped-clamped microbeam is used to track the frequency shift in real time and hence, the amount of added mass attached on the surface of the sensor until reaching a specific threshold to trigger a switch. The clamped-clamped microbeam is coated with HKUST-1 MOF, which is a very sensitive chemical layer. We demonstrated that frequency shift can be tracked in nonlinear regime using the linearly fitted upper branch in hardening behavior. The quantity of mass attached on the microbeam can be approximated using the calculated responsivity of the microbeam. Two concepts of switches triggered by mass detection were demonstrated based on the jumps a resonator experiences in a hardening or a softening behavior. Such switches are promising for applications where immediate actions, such as alarming, are needed upon detection of certain dangerous gases. To enhance the sensitivity of detection, the resonators need to be scaled further down to the sub-micron and nano scale. Noise issues however are expected to become more important since the signal-to-noise ratio in the nano scale becomes too weak.

## Figures and Tables

**Table 1:** The geometrical properties of the microbeam.

Symbol	Quantity	Dimensions
*L*	Length	600 μm
*h*	Thickness	6.85 μm
*b*	Width	50 μm
*d*	Gap	2 μm

**Figure A1:**
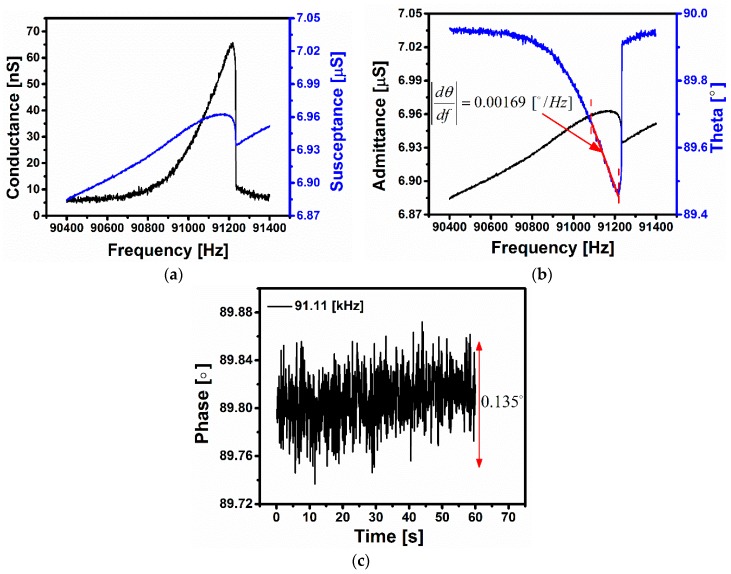
Noise analysis at a voltage load of *V_DC_* = 3 V and *V_AC_* = 1 V and at a pressure of 220 mTorr. (**a**) Conductance and susceptance as a function of the frequency near resonance; (**b**) Admittance and phase as a function of the frequency near resonance; (**c**) Variation of the phase in time at *f* = 91.11 kHz.

## Appendix A

In order to determine the minimum detectable frequency, and hence, the minimum detectable mass of the gas sensor at a given temperature, we performed a noise analysis in the case of jump-down switch [7]. The frequency shift due to thermal fluctuations around the resonator can be related to the phase variation at a given frequency. A precision impedance analyzer Agilent 4294A (Agilent Technologies Inc., San Francisco, CA, USA) which is connected to a PC with a GPIB interface from National Instruments for data acquisition has been used to characterize the microbeam electrically. The conductance and phase of the sensor are plotted in Figure A1a,b, respectively. The constant excitation frequency is selected to be in the middle of the linearly fitted zone in the phase response curve in Figure A1b, where the slope is found to be equal to $\left|d\phi /df\right|=0.00169^{\circ }/\mathrm{Hz}$.

The phase evolution as a function of time at a fixed frequency has been shown in Figure A1c. The phase noise has been calculated to be $d\phi =0.135^{\circ }$, which leads to a frequency shift $\delta f_{noise}=d\phi /\left|d\phi /df\right|=79.88\text{ Hz}$.

In order to determine the linear relation between the induced frequency shift and the added mass, we calculate the responsivity of the sensor, which can be expressed as [7]

(A1) $${ℜ}^{-1}=\left|\frac{dm}{df}\right|=\frac{2m_{eff}}{f_{res,0V}}$$

where *f_res,_*_0*V*_ = 86.8 kHz is the natural resonant frequency at *V_DC_* = 0 V and $m_{eff}$ is the effective mass of the sensor represented as *m_eff_* = *α m*, where *α* = 0.3965 for the first natural frequency of a clamped-clamped microbeam [49]. The mass of the microbeam is *m* = 510 ng. From Equation (A1), the responsivity of the sensor is ${ℜ}_{1st}^{-1}=4.65\text{ pg}/\text{Hz}$.

Based on the calculated responsivity value, one can calculate the minimum detectable mass $\delta m_{noise}$ from the minimum detectable frequency *δf_noise_* due to thermal fluctuations. Based on Equation (A1), the relation between *δm_noise_* and *δf_noise_* can be written as

(A2) $$\delta m_{noise}={ℜ}^{-1}\delta f_{noise}$$

The minimum detectable mass from Equations (A1) and (A2) is equal to *δm_noise_* = 371.442 pg.
